# P-774. High-Bioavailability vs Low-Bioavailability Beta-Lactams in the Treatment of Lower Urinary Tract Infections in Adult and Pediatric Patients

**DOI:** 10.1093/ofid/ofaf695.985

**Published:** 2026-01-11

**Authors:** Kenina Silvera, Sarah Piccuirro, Denise Kelley, Clayton Lambert

**Affiliations:** Ascension Seton, Austin, Texas; Ascension Seton, Austin, Texas; Ascension Seton, Austin, Texas; Ascension Seton, Austin, Texas

## Abstract

**Background:**

With increasing use of beta-lactams for UTIs, there is the concern for inferior efficacy of low-bioavailability agents due to their comparatively low bioavailability and higher degrees of protein binding resulting in inadequate target attainment of percent of time above the MIC. The purpose of this study is to evaluate rates of treatment failure for lower UTIs with high-bioavailability beta-lactams (HBBLs) compared to low-bioavailability beta-lactams (LBBLs) in patients seen and discharged from the emergency department (ED).

**Methods:**

This is an ongoing multicenter, retrospective cohort study evaluating rates of treatment failure for lower UTIs in patients prescribed HBBLs (amoxicillin/clavulanate and cephalexin) versus LBBLs (cefuroxime, cefpodoxime, and cefdinir) between January 2018-December 2024. Patients were included if they presented to an Ascension Seton ED with urinary symptoms and were discharged from the ED with diagnosis of UTI and a prescription for one of the target antibiotics. Patients were excluded if presenting with systemic signs or symptoms of infection, multiple infectious diagnoses on discharge requiring antibiotic therapy, lack of urine culture, negative or contaminated urine culture, or urinary isolate identified as resistant to the prescribed antibiotic. Treatment failure was defined as either a) return to an Ascension Seton facility with urinary symptoms and diagnosis of UTI requiring additional therapy, or b) receipt of a prescription for another antibiotic within this period.

**Results:**

A total of 233 patients have been included, with 107 prescribed HBBLs, 56 receiving amoxicillin/clavulanate and 51 cephalexin, and 126 prescribed LBBLs, 47 receiving cefuroxime, 23 cefpodoxime, and 56 cefdinir. Of the 233 patients, 38 (16.3%) are < 18 years old. Treatment failure at 14 days occurred in 6 patients (5.6%) in the HBBL group and 5 patients (4%) in the LBBL group. At 28 days, treatment failure occurred in 12 patients in the HBBL group (11.2%) and 16 patients in the LBBL group (12.7%).

**Conclusion:**

In this preliminary analysis, there are no significant rates of treatment failure at 14 or 28 days for HBBLs versus LBBLs in the treatment of lower urinary tract infections.

**Disclosures:**

All Authors: No reported disclosures

